# Effect of CMV and Aging on the Differential Expression of CD300a, CD161, T-bet, and Eomes on NK Cell Subsets

**DOI:** 10.3389/fimmu.2016.00476

**Published:** 2016-11-07

**Authors:** Nelson Lopez-Sejas, Carmen Campos, Fakhri Hassouneh, Beatriz Sanchez-Correa, Raquel Tarazona, Alejandra Pera, Rafael Solana

**Affiliations:** ^1^Maimonides Biomedicine Institute of Cordoba (IMIBIC), Reina Sofia Hospital, University of Cordoba, Cordoba, Spain; ^2^Immunology Unit, Department of Physiology, University of Extremadura, Cáceres, Spain

**Keywords:** aging, CMV, CD57, CD161, CD300a, Eomes, NK cell subsets, T-bet

## Abstract

Natural killer (NK) cells are innate lymphoid cells involved in the defense against virus-infected cells and tumor cells. NK cell phenotype and function is affected with age and cytomegalovirus (CMV) latent infection. Aging affects the frequency and phenotype of NK cells, and CMV infection also contributes to these alterations. Thus, a reduction of CD56^bright^ NK cell subpopulation associated with age and an expansion of memory-like NK cells CD56^dim^CD57^+^NKG2C^+^ probably related to CMV seropositivity have been described. NK cells express T-bet and Eomes transcription factors that are necessary for the development of NK cells. Here, we analyze the effect of age and CMV seropositivity on the expression of CD300a and CD161 inhibitory receptors, and T-bet and Eomes transcription factors in NK cell subsets defined by the expression of CD56 and CD57. CD300a is expressed by the majority of NK cells. CD56^bright^ NK cells express higher levels of CD300a than CD56^dim^ NK cells. An increase in the expression of CD300a was associated with age, whereas a decreased expression of CD161 in CD56^dim^ NK cells was associated with CMV seropositivity. In CD56^dim^ NK cells, an increased percentage of CD57^+^CD300a^+^ and a reduction in the percentage of CD161^+^CD300a^+^ cells were found to be associated with CMV seropositivity. Regarding T-bet and Eomes transcription factors, CMV seropositivity was associated with a decrease of T-bet^hi^ in CD56^dim^CD57^+^ NK cells from young individuals, whereas Eomes expression was increased with CMV seropositivity in both CD56^bright^ and CD56^dim^CD57^+/−^ (from middle age and young individuals, respectively) and was decreased with aging in all NK subsets from the three group of age. In conclusion, CMV infection and age induce significant changes in the expression of CD300a and CD161 in NK cell subsets defined by the expression of CD56 and CD57. T-bet and Eomes are differentially expressed on NK cell subsets, and their expression is affected by CMV latent infection and aging.

## Introduction

Natural killer (NK) cells are lymphocytes of innate immune response responsible for killing virus-infected cells and tumor cells. Frequency, phenotype, and function of NK cell subsets change in aging, and these changes are considered part of a general process of age-associated immune dysfunctions defined as immunosenescence (1–4). Immunosenescence affects adaptive and innate immunity, and it is associated with increased incidence and severity of infections and decreased response to vaccination (5–11).

It has been shown that percentage of CD56^dim^ NK cell subset (the main subset of NK cells and most cytotoxic) is increased (12, 13) or maintained (14) by age, whereas percentage of immature CD56^bright^ NK cells is decreased (12–15). On the other hand, the expression of different receptors on NK cells is also altered in aging (16–18). It has been found a decrease in the expression of natural cytotoxicity receptors (such as NKp30 and NKp46) and activating receptor DNAM-1 on CD56^dim^ NK subset (3, 19–21) an increased expression of CD57 (18, 20, 22), as well as reciprocal changes in NKG2A and killer immunoglobulin-like (KIR) inhibitory receptors, associated with age (18, 20, 22).

Human cytomegalovirus (CMV) chronic infection is related to a deterioration of the immune system that affect adaptive and innate immunity, and it has been postulated that CMV infection is a major driving force contributing to immunosenescence (10, 23, 24). CMV is a human herpesvirus type 5 (HHV5), which replicate in different cell types. Although CMV seropositivity is influenced by geographic, ethnic, and socio-economical factors, it has been shown that it increases with age in all populations studied (25). CMV prevalence is very high in Spain and more than 80% of individuals over the age of 40 years are CMV-seropositive (26).

Cytomegalovirus seropositivity can reshape the repertoire of NK cells (2, 27), particularly with an expansion of NKG2C^+^ NK cells, which also coexpress CD57 marker (14, 28, 29). Recent studies stratifying donors according to CMV serology have shown that increase of CD57 expression on CD56^dim^ NK cells is related to CMV seropositivity rather than aging (14) as well as the decreased expression of other surface receptors of NK cells, such as NKp30 (30) or CD161 (31). Thus, aging is associated with a loss of CD56^bright^ NK cell subpopulation (probably due to a decrease in the production of new NK cells in the bone marrow) and an expansion of memory-like NK cells CD56^dim^CD57^+^NKG2C^+^ that is mainly related to CMV seropositivity [reviewed in Ref. (2)].

Although it is well established that aging and CMV infection are associated with changes in NK cells, including alterations in the expression of activating (e.g., NCRs, NKG2C, and DNAM-1) and inhibitory receptors (e.g., KIR and NKG2A), little is known on the effect of aging and CMV on the expression of other inhibitory receptors such as CD300a and CD161 in NK cell subpopulations. CD300a is an inhibitory receptor expressed by NK cells that belongs to the CD300 family of molecules. These receptors are broadly expressed on immune cells and modulate their function *via* paired activating and inhibitory receptors that recognize lipids exposed on the plasma membrane of dead and activated cells including aminophospholipids such as phosphatidylserine (PS) and phosphatidylethanolamine (PE) (32). The analysis of its expression can be used in diagnosis and therapy in several pathological situations including infectious diseases, allergy, or cancer [for review, see Ref. (33)]. The human CD161 inhibitory receptor (also termed NKR-P1A, KLRB1, and CLEC5B) was originally described as a disulfide-linked homodimer of the C-type lectin superfamily expressed on subsets of NK cells and T lymphocytes (34) that binds the lectin-like transcript 1 (LLT1, also named CLEC2D, OCIL, and CLAX) (35, 36). The binding of CD161 on NK cells with its ligand on target cells results in inhibition of NK cell cytotoxicity by a mechanism involving the activation of acid sphingomyelinase (37). CD161 can also be expressed on subsets of other cells of the immune system, and different functional capacities have been shown after the interaction with its ligand, which can be upregulated during the immune response and during pathological circumstances. The current knowledge of NKRP1 receptors and their genetically linked CLEC2 ligand in human and other species has been recently reviewed (38, 39).

Natural killer cells are included in group 1 of the innate lymphoid cell (ILC), characterized by the release of interferon-gamma (IFN-γ) upon stimulation, and by the expression of T-bet and eomesodermin (Eomes) transcription factors (40–42). Both T-bet and Eomes are constitutively expressed by murine (43) and human (44, 45) NK cells and are necessary for the proper development of NK cells (46), sharing several functions. It has been observed that the frequency of T-bet^+^ cells and the level of T-bet expression per cell is significantly greater in the CD56^dim^ population compared to the CD56^bright^ population from peripheral human immune cells, contrary to Eomes expression pattern, suggesting the existence of a relationship among the expression levels of both transcription factors and the functionality of these cells (45). Thus, T-bet is related to terminal stages of maturation, while Eomes is downregulated during peripheral maturation (47).

Considering that aging affects the frequency and phenotype of NK cells and that CMV infection contributes to age-associated changes in NK cells; in this work, we have analyzed the effect of age and CMV seropositivity on inhibitory receptors CD300a and CD161 in NK cell subpopulations. Additionally, we have investigated the effect of age and CMV infection on T-bet and Eomes transcription factors expression in the CD56^bright^CD57^−^, CD56^dim^CD57^−^, and CD56^dim^CD57^+^ NK cell subsets.

## Materials and Methods

### Study Subjects

A total of 72 healthy adults voluntarily participated in the study, stratified according to age: 18–35 years (young), 40–65 years (middle age), and >70 years (old). Young and middle age donors were further divided according to CMV serology (CMV-seropositive and CMV-seronegative). However, all elderly donors included in the study were CMV-seropositive, given that the prevalence of CMV seropositivity in Spain in individuals over the age of 40 years is 80% (26) and, in our geographic area (Andalusia, Southern Spain), about 99% of individuals over 65 years are CMV-seropositive. Thus, the absence of a group of CMV-seronegative old donors represents a limitation of the study, making difficult to isolate age-related effect from the effect of chronic CMV infection in elderly individuals.

All donors were informed and signed informed consent to participate in the study and were included according to following inclusion criteria: no infection at the time of extraction, not suffer or have suffered cancer or autoimmune diseases, and not be under immunosuppressive drugs or calcium channel blockers. The study was approved by the Ethics Committee of Hospital Universitario Reina Sofia of Córdoba (Spain).

### Sample Collection and Processing

Peripheral blood mononuclear cells (PBMCs) were obtained from blood samples (collected in lithium heparin tubes) and isolated by density gradient centrifugation using Ficoll Histopage-1077 (Sigma-Aldrich, St. Louis, MO, USA). Aliquots of cells were cryopreserved in FBS (Sigma-Aldrich, St Louis, MO, USA) with 10% DMSO (Panreac Chemistry SAU, Barcelona, Spain) in cryotubes at concentrations 5–6 × 10^6^ cells/mL until further use.

A sample of plasma or serum was retrieved from all donors to analyze CMV-specific IgG and IgM. CMV serology was determined by using automated enzyme-linked immunosorbent assay (ELISA) (DRG International, Mountainside, NY, USA).

### Analysis of NK Cell Receptors by Flow Cytometry

For surface marker analysis and computation of frequency of cells expressing CD57, CD161, or CD300a, cryopreserved PBMCs were used. Cell thawing was carried out in RPMI 1640 (Sigma-Aldrich) with 20% FBS (Gibco, Life Technologies California, USA). For flow cytometry staining, the following antibodies (mAbs) were used: anti-CD3 PerCP (clone: BW 264/56, Miltenyi Biotec), anti-CD56 PE-Cy7 (clone: B159, BD Pharmingen), anti-CD57 VioBlue (clone: TB03, Miltenyi Biotec), anti-CD300a PE (clone: E59.126, Beckman Coulter), and anti-CD161 APC (clone: DX12, BD Pharmingen). Cells were acquired on a MACSQuant cytometer (Miltenyi Biotec, Bergisch Gladbach, Germany). Compensation for flow cytometry was performed using single cell staining.

Data were analyzed using FlowJo v10 (Tree Star, Inc.). CD3^−^CD56^+^ NK cells were gated from total peripheral blood lymphocytes (PBLs), after singlets gating (Figure S1 in Supplementary Material). Two NK cell subsets were defined (CD56^bright^ and CD56^dim^) according to the level of CD56 marker expression. Subsequently, cells were gated according to the coexpression of CD57, CD161, and CD300a markers. Fluorescence minus one controls (FMO control) were used to identify and gate cells. FMO controls contain all the fluorochromes of the panel, except the one that was being measured. Analysis of coexpression of the three receptors was performed using FlowJo’s Boolean gating options.

### Transcription Factors Expression Analysis

The expression of T-bet and Eomes transcription factors was analyzed using cryopreserved PBMCs, thawed as indicated above. Surface staining was performed using anti-CD7 APC (clone: M-T701, BD Pharmingen), anti-CD56 BV421 (clone: NCAM16.2, BD Horizon), anti-CD16 PE- Vio770 (clone: VEP13, Miltenyi Biotec), anti-CD57 Biotin-Anti-Biotin-Viogreen (Miltenyi Biotec), and anti-CD3/anti-CD14/anti-CD19 conjugated with APC-Vio770 (clones: BW264/56, TÜK4, LT19 Miltenyi Biotec). After cell fixation and permeabilization using the Kit FoxP3 Staining Buffer Set (Miltenyi Biotec), following the manufacturer’s instructions, intracellular staining was realized with anti-T-bet PerCP Cy5.5 (clone: 04-46, BD Pharmingen) and anti-Eomes FITC (clone: WD1928, eBioscience) antibodies. Cells were then acquired on a 10 parameter MACSQuant cytometer (Miltenyi Biotec, Bergisch Gladbach, Germany).

Data were analyzed using FlowJo v10 (Tree Star, Inc.). Once selected the CD7^+^CD3^−^CD19^−^CD14^−^ cells from PBLs singlets, three populations of NK cells were described: CD56^bright^CD57^−^, CD56^dim^CD57^−^, and CD56^dim^CD57^+^. Then, the intracellular expression of T-bet and Eomes was measured in each of these three subpopulations (Figure S2 in Supplementary Material). FMO controls and flow cytometry compensation were performed as indicated above.

### Processing of Data and Statistical Analysis

For statistical analysis, Shapiro–Wilk normality test was performed for different groups. Since the distribution of measured values was not normal, the groups were evaluated by the non-parametric Kruskal–Wallis test (for comparison multiple) and Mann–Whitney test (for comparison sample pairs). Non-parametric Friedman test (for comparison multiple) and Wilcoxon test were used to test for differences between groups when the samples are related. The results are shown in graphs with interquartile medium range using GraphPad Prism (version 5.0), and analysis of Boolean gating data was performed by SPICE 5.35 software (Mario Roederer, ImmunoTechnology Section, Vaccine Research Centre, NIH, Bethesda, MD, USA; http://www.niaid.nih.gov) (48). To compare the pie charts, we used SPICE’s permutation analysis, which asks how often the samples that comprise two different pies charts, would be different simply by chance (10,000 permutations). All statistical analysis was performed with SPSS 18.0 (SPSS Inc., Chicago, IL, USA). *p*-Values <0.05 were considered significant.

## Results

### Increase of CD300a and Decrease of CD161 Expression from CMV-Seropositive Old Individuals

**Figure 1:**
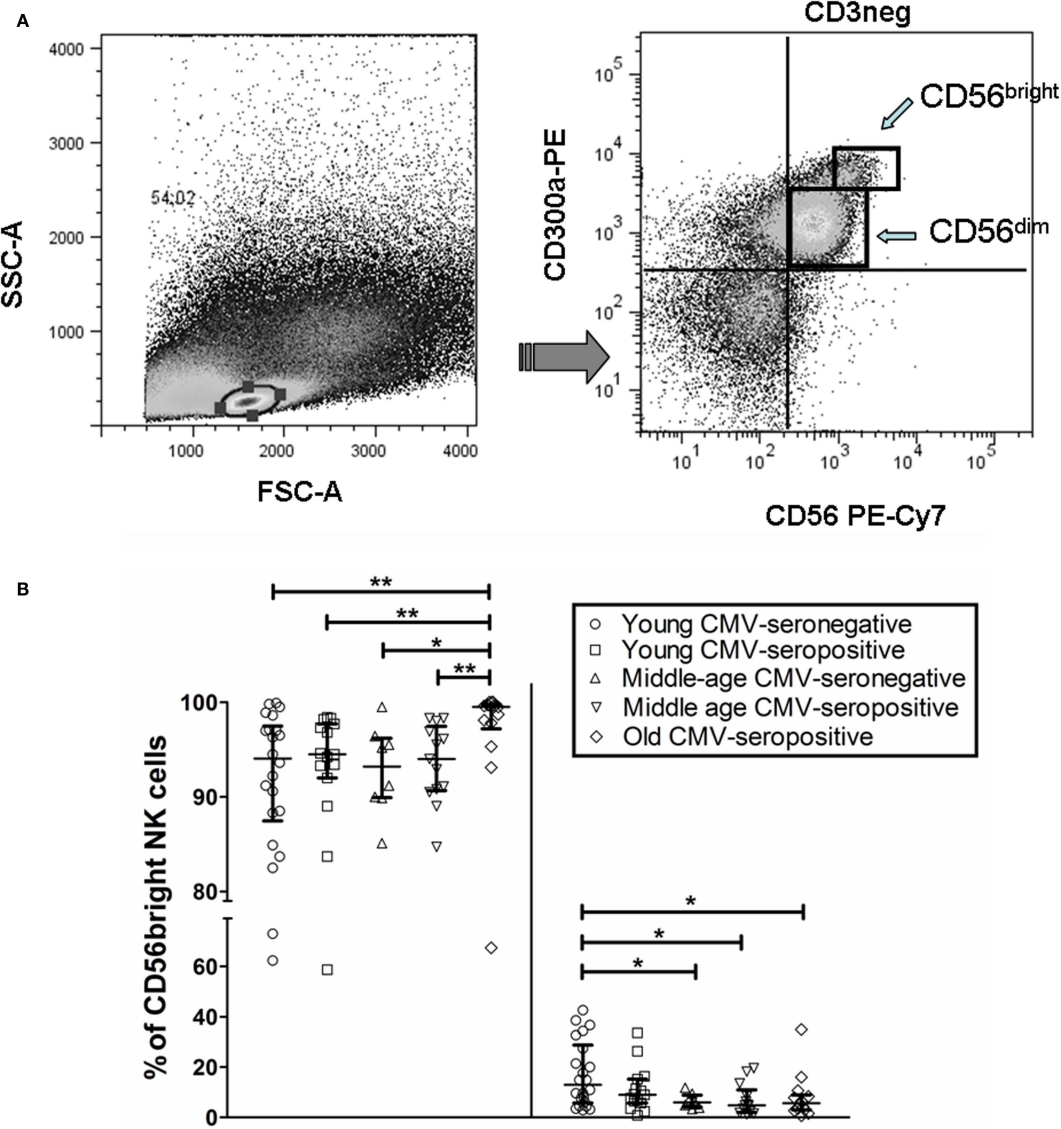

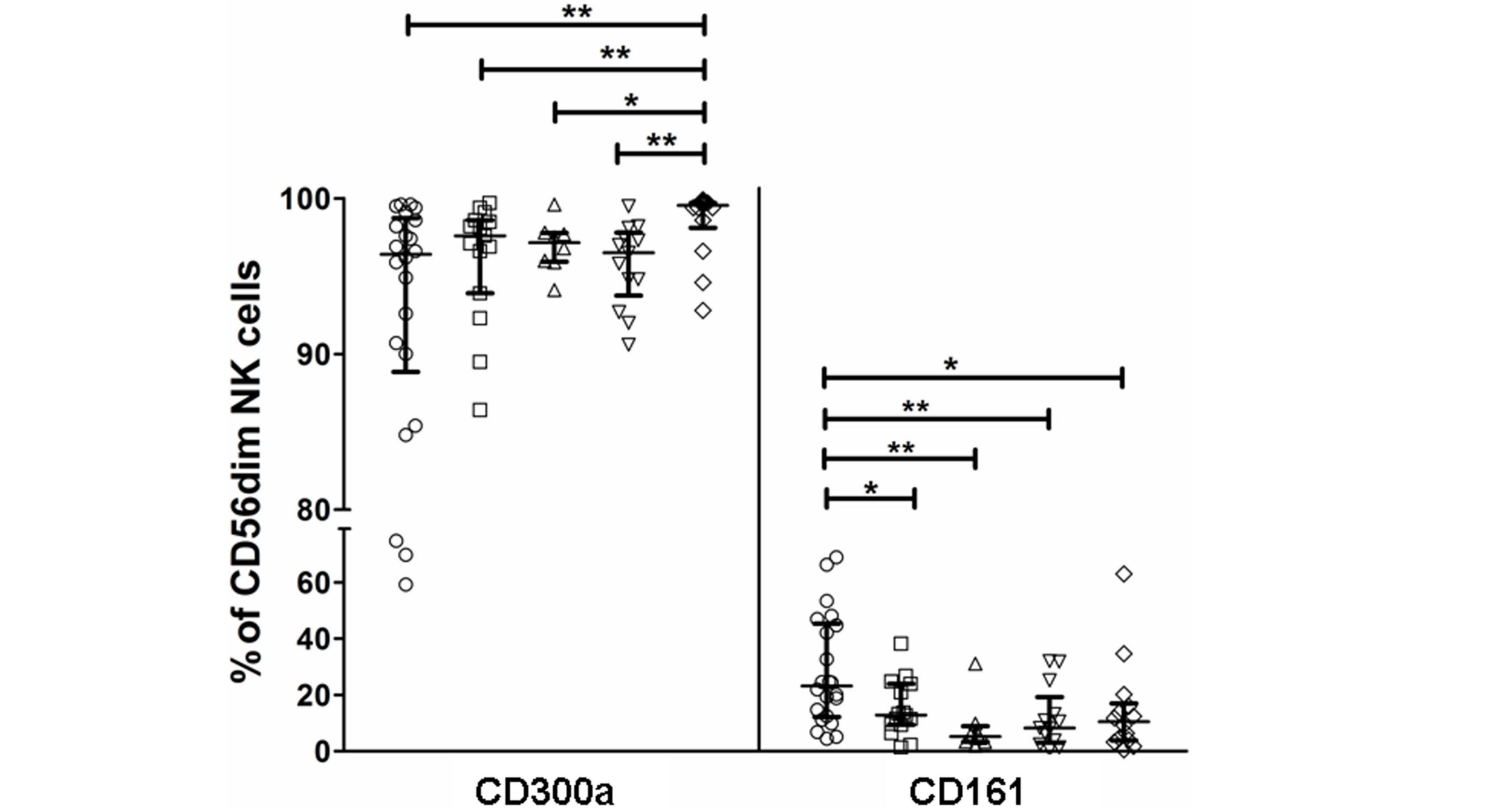
**CD300a and CD161 expression on CD56^dim^ and CD56^bright^ NK cells from young, middle age, and old individuals, according to CMV seropositivity**. **(A)** Differential expression of CD300a on NK cell subsets. NK cells were defined as CD3^−^CD56^+^ and characterized by CD300a expression as CD56^bright^CD300a^hi^ and CD56^dim^CD300a^lo^. **(B)** Effect of age and CMV seropositivity on the expression of CD300a and CD161. Expression (percentage) of CD300a and CD161 markers was determined on NK cell subsets from young CMV-seronegative (*n* = 22), young CMV-seropositive (*n* = 15), middle age CMV-seronegative (*n* = 8), middle age CMV-seropositive (*n* = 13), and old CMV-seropositive donors (*n* = 14). CD56^+^CD3^−^ NK cells were gated from singlets peripheral blood lymphocytes (PBLs). Then, two NK cell subsets were defined (CD56^bright^ and CD56^dim^) according to the level of CD56 marker expression, and cells were gated according to the expression of CD57, CD161, and CD300a markers. Non-parametric Kruskal–Wallis test (for multiple comparisons) and Mann–Whitney test (for paired comparisons) was used. Graphs showed the median with interquartile range, and results were considered significant at *p < 0.05, **p < 0.01, and ***p < 0.001.

We analyzed by flow cytometry the expression of CD300a and CD161 inhibitory receptors on NK cells (CD56^bright^ and CD56^dim^) from healthy individuals stratified by age and CMV latent infection. Our data showed that both receptors exhibit a differential expression pattern. Particularly, the majority of CD56^dim^ and CD56^bright^ NK cells expressed CD300a, whereas CD161 was expressed on a subpopulation of NK cells regardless of the NK cell subset analyzed (Figure S1 in Supplementary Material). Moreover, when we gated the NK cell subsets according to CD56 and CD300a (referred to CD3^−^ cells), we observed that the majority of CD56^bright^ NK cells were CD300a^hi^, whereas a high percentage of CD56^dim^ NK cells were CD300a^lo^ (Figure 1A). Analysis of the effect of age and CMV seropositivity on the expression of these receptors by NK cell subsets (gating strategy in Figure S1 in Supplementary Material) showed an increase of CD300a on CD56^bright^ and CD56^dim^ subsets from CMV-seropositive old individuals compared with middle-aged and young donors (independently of CMV seropositivity) (Figure 1B).

The analysis of CD161 expression on CD56^dim^ NK cells showed that young CMV-seronegative donors expressed higher levels of CD161 than young CMV-seropositive or middle age CMV-seronegative individuals, supporting that CMV and age independently associated with decreased expression of CD161 in this NK cell subset. On the contrary, the expression of CD161 on CD56^bright^ NK cells was not affected by CMV seropositivity in young donors, whereas it was decreased in middle age CMV-seronegative individuals. The expression of CD161 was lower on both NK cell subsets in middle age and old CMV-seropositive donors compared with young CMV-seronegative individuals (Figure 1B).

These results indicate an expansion of CD300a^+^ on NK cell subsets (CD56^bright^ and CD56^dim^) from healthy old individuals (all CMV-seropositive), likely associated with the combined effect of CMV infection and age, and a decrease of CD161^+^ NK cells related to CMV seropositivity (CD56^dim^ NK cells) and age (CD56^bright^ and CD56^dim^ NK cells).

In this analysis, we have also observed an increased percentage of CD56^dim^CD57^+^ NK cells associated with CMV seropositivity. Also, CD56^bright^ NK cells do not express or express very low levels of CD57 on their surface (data not shown).

### Coexpression of CD300a, CD161, and CD57 on NK Cell Subsets

**Figure 2:**
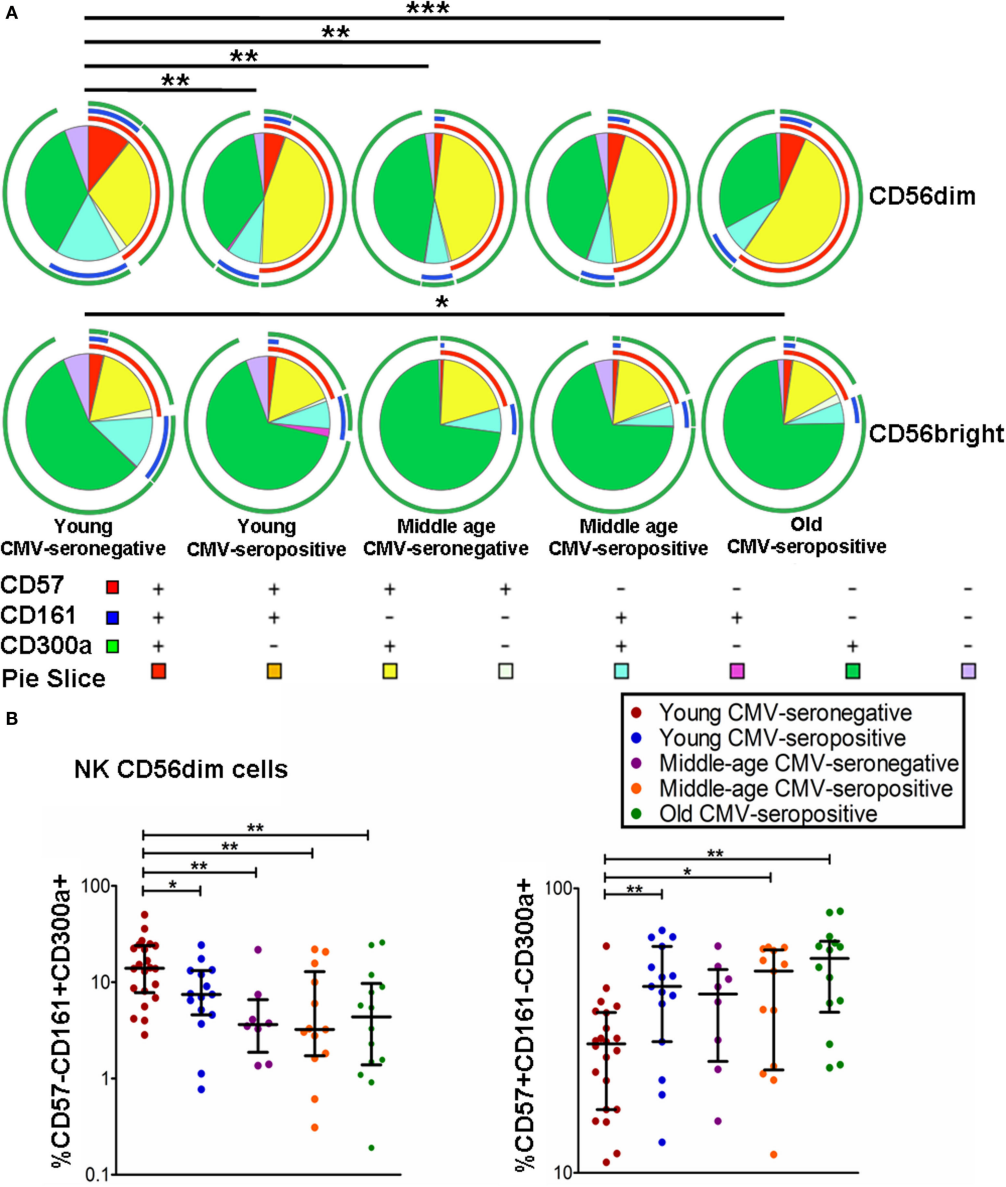

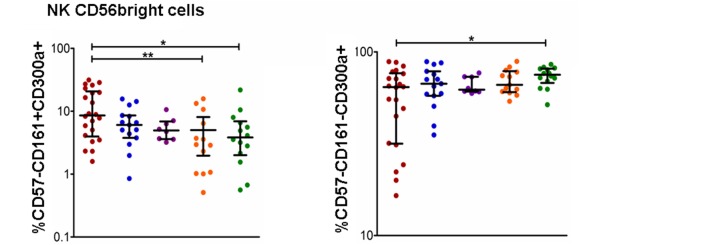
**Coexpression of CD57, CD161, and CD300a in NK cell subsets**. **(A)** CD57, CD161, and CD300a expression profile (pie charts) in CD56^dim^ and CD56^bright^ NK cells from 72 healthy individuals stratified by age and CMV serostatus. Analysis of coexpression of three receptors was performed using FlowJo’s Boolean gating options. For analysis of complex multivariate datasets after of flow cytometry, we used the application simplified presentation of incredibly complex evaluations (SPICE). **(B)** Graphs show the coexpression of CD57/CD161/CD300a NK subsets in which we found statistical differences among the four groups studied. Non-parametric Kruskal–Wallis test (for multiple comparisons) and Mann–Whitney test (for paired comparisons) were used. Graphs showed the median with interquartile range. Results were considered significant at *p < 0.05, **p < 0.01, and ***p < 0.001.

The coexpression of CD300a, CD161, and CD57 was measured on CD56^dim^ and CD56^bright^ NK cells using FlowJo’s Boolean gating options. The majority of CD56^bright^ and CD56^dim^ NK cells expressed CD300a on their surface, either alone or in combination with CD161 or CD57. A minor subset of CD300a^+^ NK cells coexpressed CD161 and CD57 (Figure 2A).

The results on the effect of age and CMV seropositivity on the coexpression of CD300a, CD161, and CD57, revealed that CMV seropositivity, but not age, was associated with an increase of CD56^dim^ NK cells coexpressing CD300a and CD57. We also observed a decrease of CD56^dim^CD300a^+^CD161^+^ NK cells related to both CMV seropositivity and age. No significant differences were found in the percentage of CD56^dim^CD300a^+^CD57^−^CD161^−^ NK cells among the different groups studied (data not shown). On the other hand, we have also observed an increase of CD56^bright^CD300a^+^CD57^−^CD161^−^ NK cells and a decrease of CD56^bright^ coexpressing CD300a and CD161 in old individuals (Figures 2A,B).

### Different Expression Patterns of T-bet and Eomes Transcription Factors in NK Cell Subsets

We analyzed the expression of T-bet and Eomes transcription factors in three subpopulations of NK cells, according to the expression of CD56, CD16, and CD57 markers: CD56^bright^CD16^+/−^CD57^−^, CD56^dim^CD16^+^CD57^−^, and CD56^dim^CD16^+^CD57^+^ NK cells (Figure S2 in Supplementary Material). Although Eomes was expressed in most NK cells in all groups studied, the analysis of its expression pattern showed higher levels in the most immature NK cells (CD56^bright^CD16^+/−^CD57^−^) with a decline in its expression in the CD56^dim^CD16^+^CD57^−^ and in the most differentiated NK cells (CD56^dim^CD16^+^CD57^+^) (Table 1).

**Table 1:** **Eomes expression (MFI) in NK subsets from donors stratified according to age and CMV seropositivity**.

	CD56^bright^CD16^−^CD57^−^ (1)	CD56^dim^CD16^+^CD57^−^ (2)	CD56^dim^CD16^+^CD57^+^ (3)	ρ	ρ (1–2)	ρ (2–3)	ρ (1–3)
Young CMV^−^	3.47 (4.46–2.58)^a^	2.12 (2.23–1.35)	1.59 (1.78–0.84)	0.000	0.005	0.005	0.005
Young CMV^+^	3.75 (4.91–2.35)	2.28 (2.67–1.9)	2.18 (2.34–1.58)	0.007	0.007	0.169	0.007
Middle age CMV^−^	3.48 (4.74–2.33)	1.87 (2.41–1.65)	1.45 (2.4–1.28)	0.001	0.018	0.018	0.018
Middle age CMV^+^	3.32 (4.54–2.96)	2.15 (2.52–1.67)	1.6 (2.1–1.41)	0.000	0.008	0.008	0.008
Old CMV^+^	3.02 (3.79–2.46)	2.01 (2.71–1.59)	1.8 (2.05–1.2)	0.000	0.005	0.005	0.005

*^a^Values expressed as median (interquartile range, 75–25)*.

*ρ value obtained by the Friedman rank sum test*.

*ρ values comparing (1–2), (2–3), and (1–3) groups obtained by the Wilcoxon test*.

The expression of T-bet in NK cell subsets showed a gradient of expression and two subsets can be distinguished: T-bet^hi^ and T-bet^int^ NK cells. The percentage of NK cells expressing T-bet^hi^ was higher than the percentage of NK cells expressing T-bet^int^ in the three subsets studied (Figure S2 in Supplementary Material). The analysis of T-bet MFI in the different NK cell subsets showed that T-bet expression was lower in CD56^bright^CD16^+/−^CD57^−^ NK cells from each group studied than in CD56^dim^CD16^+^CD57^−^ and CD56^dim^CD16^+^ CD57^+^ NK cells into the same group (Table 2).

**Table 2:** **T-bet expression (MFI) in NK subsets from donors stratified according to age and CMV seropositivity**.

	CD56^bright^CD16^−^CD57^−^ (1)	CD56^dim^CD16^+^CD57^−^ (2)	CD56^dim^CD16^+^CD57^+^ (3)	ρ	ρ (1–2)	ρ (2–3)	ρ (1–3)
Young CMV^−^	6.31 (10.2–2.91)^a^	8.84 (12.6–5.89)	9.31 (12.5–6.24)	0.000	0.008	0.022	0.005
Young CMV^+^	7.17 (10.3–3.47)	10.84 (12.2–7.26)	9.64 (11.4–7.49)	0.000	0.005	0.011	0.008
Middle age CMV^−^	10.10 (13–1.89)	12.8 (13.8–5.77)	13.2 (13.8–5.84)	0.004	0.043	0.042	0.018
Middle age CMV^+^	7.09 (10.8–3.85)	10.3 (13.5–6.97)	10.7 (13.9–6.79)	0.003	0.008	0.285	0.011
Old CMV^+^	4.85 (11.3–2.79)	7.57 (11.6–6.42)	7.22 (10.6–5.95)	0.007	0.017	0.013	0.114

*^a^Values expressed as median (interquartile range, 75–25)*.

*ρ value obtained by the Friedman rank sum test*.

*ρ values comparing (1–2), (2–3), and (1–3) groups obtained by the Wilcoxon test*.

**Figure 3:**
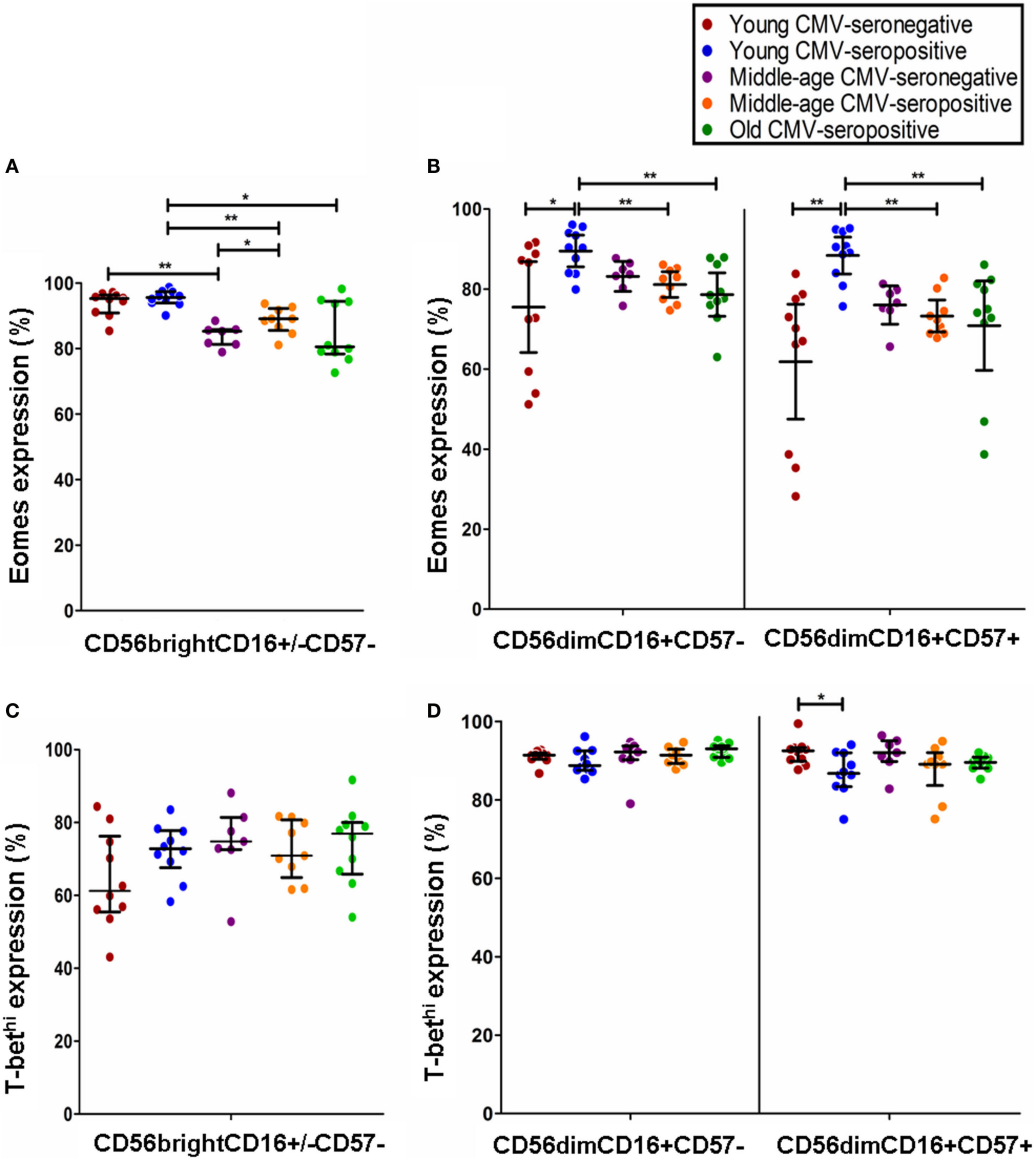
**Effect of age and CMV seropositivity on Eomes and T-bet expression**. The expression of T-bet and Eomes was measured in CD56^bright^CD16^+/−^CD57^−^, CD56^dim^CD16^+^CD57^−^, and CD56^dim^CD16^+^CD57^+^ NK cells from 46 healthy individuals (10 young CMV-seronegative, 10 young CMV-seropositive, 7 middle age CMV-seronegative, 9 middle age CMV-seropositive, and 10 old CMV-seropositive donors). **(A)** Analysis of Eomes expression in CD56^bright^CD16^+/−^CD57^−^ NK cells. **(B)** Analysis of Eomes expression in CD56^dim^CD16^+^CD57^−^ and CD56^dim^CD16^+^CD57^+^ NK cells **(C)** Analysis of T-bet expression in CD56^bright^CD16^+/−^CD57^−^ NK cells. **(D)** Analysis of T-bet expression in CD56^dim^CD16^+^CD57^−^ and CD56^dim^CD16^+^CD57^+^ NK cells. Non-parametric Kruskal–Wallis test (for multiple comparisons) and Mann–Whitney test (for paired comparisons) were used. Graphs showed the median with interquartile range. Results were considered significant at *p < 0.05, **p < 0.01, and ***p < 0.001.

The analysis of Eomes expression in NK cell subsets from CMV-seronegative and CMV-seropositive individuals showed that the percentage of positive cells was significantly increased in CD56^bright^CD16^+/−^CD57^−^ NK cells from CMV-seropositive middle age donors and in CD56^dim^CD16^+^CD57^+^ and CD56^dim^CD16^+^CD57^−^ from CMV-seropositive young donors compared with the CMV-seronegative counterparts (Figures 3A,B). The analysis of the effect of age on Eomes expression showed a decreased expression in CD56^bright^CD16^+/−^CD57^−^ NK cells from CMV-seronegative middle age donors compared with CMV-seronegative young individuals (Figure 3A). Eomes expression in CMV-seropositive donors was significantly higher in the young compared with the middle age and old groups in the three NK cell subsets considered (Figures 3A,B).

The effect of age and CMV seropositivity on T-bet expression was also analyzed. The results only showed a significant decrease in the percentage of T-bet^hi^ within the CD56^dim^CD16^+^CD57^+^ NK cell subset from CMV-seropositive compared with CMV-seronegative young donors (Figures 3C,D).

## Discussion

Cumulative evidences in the last decade support that aging and CMV latent infection combine to influence the immune phenotype and function of immune cells, including NK cells, in different ways that have been often overlooked in studies aiming to analyze the effect of aging on NK cells without considering the CMV serostatus of the individuals studied.

In this work, we have analyzed the effect of CMV seropositivity and aging on the expression of CD300a and CD161 receptors and transcription factors T-bet and Eomes on peripheral blood NK cell subsets with different levels of maturation.

Our results show that CD57^+^CD56^dim^ NK cells are expanded in CMV-seropositive individuals and that these cells are not further expanded by aging. In addition, the majority of these cells also coexpress CD300a, but not CD161. The expansion of CD57^+^CD56^dim^ NK cells support previous data showing that acute and latent CMV infection leads to the expansion of CD57^+^CD56^dim^ NK cells that also express NKG2C^+^ (28, 49–54). CMV seropositivity is also associated with a decreased expression of other NK receptors, in some cases, as a consequence of the shift of NK cells to the more differentiated NKG2C^+^CD57^+^ phenotype (14, 27, 30, 54). These cells have some characteristics of adaptive immunity and are considered “memory” or “adaptive” NK cells (55–58). The magnitude of the expansion of NKG2C^high^ NK cells is determined by the magnitude of the proinflammatory cytokine secretion upon NK cell activation (59), and it has been proposed that these cells contribute to the proinflammatory environment based on the relation between the percentage of NKG2C^+^ cells, elevated levels of PCR, and cardiovascular risk determinants of CMV-seropositive individuals (60, 61).

Whereas it has been show that both aging and CMV infection are associated with a decreased expression of several NK activating receptors (with the exception of NKG2C that is increased in CMV-seropositive individuals), their effect on the expression of inhibitory receptors is still controversial. Thus, whereas different studies have shown an age-associated increased expression of KIR (20, 62) and a decreased expression of NKG2A (20), others have not found significant differences in the expression of KIR or NKG2A in the elderly (14, 17, 18, 30) or even a decreased expression of KIR (CD158a) in middle age donors if they are CMV-seropositive (63). The expression of another inhibitory receptor KLRG-1 is significantly reduced with aging (21) and with CMV seropositivity in the young individuals (63).

The inhibitory receptor CD300a is expressed on the majority of NK cells, and its expression increases with aging in CMV-seropositive individuals both in CD56^bright^ and CD56^dim^ NK cell subsets. As stated in Section “Materials and Methods,” in our geographic area (Andalusia, Southern Spain), about 99% of individuals over 65 years are CMV-seropositive. Thus, the high prevalence of CMV in our geographic area supposes a limitation of the study as we lack of a group of CMV-seronegative elderly donors. This limitation makes difficult to assess the differential effect of CMV and aging when we observe changes only in the group of old CMV-seropositive individuals compared with the other groups. This is the case of the higher expression of CD300a found in old CMV-seropositive individuals compared with the other groups studied. Since we do not observe an effect of CMV seropositivity between in the young and middle age groups, it could be thought that the higher expression of CD300a found in old CMV-seropositive individuals is likely due to aging. However, due to this limitation, we can only conclude that aging has an effect on CD300a expression on CMV-seropositive donors. CD300a is an inhibitory receptor that can be expressed by NK cells and that deliver inhibitory signals upon binding to PS expressed by tumor cells (64). The binding of CD300a on human or porcine NK cells to the surface of the pseudorabies virus porcine infected cell is increased by the US3 protein kinase of this alpha-herpesvirus in an aminophospholipids and p21-activated kinases dependent way, providing protection of infected cells against NK cell cytotoxicity (65). These results support the possible relevance of CD300a as a possible NK cell evasion strategy by CD300a-modulating viruses and cancer cells.

Although CD300a is highly homologous to CD300c and both receptors are considered as paired receptors with inhibitory and activating roles, respectively, very little is known on the expression and function of CD300c on NK cells. It has been recently shown that NK cells do not express (or express very low levels of) CD300c and that its expression is induced uniquely on CD56^bright^ NK cells after their treatment with IL-2 or IL-15 (66). The analysis of the interaction of CD300a and CD300c with their ligands shows differences on their binding affinity to the lipid ligands. Whereas both CD300a and CD300c show similar binding to PS, it has been shown that CD300a exhibits a stronger binding to dead cells and to PE than CD300c and PE induces a negative response of IL-2 preactivated CD56^bright^ NK cells, supporting that the inhibitory signals triggered by CD300a after binding to its lipid ligands overrides the signals triggered by its activating counterpart CD300c (66). These findings are consistent with these shown for other paired receptors such as KIR2DL1 and CD94/NKG2A that have higher binding affinity to HLA-CLys80 and HLA-E, respectively, than their activating counter parts KIR2DS1 and CD94/NKG2C. The increased expression of CD300a with aging both in CD56^bright^ and CD56^dim^ subsets can contribute together with other age-associated alterations of NK cells to the decreased functional capacity of these cells in the elderly.

The human CD161 receptor, expressed in a subset of NK cells, is an inhibitory receptor that, after interacting with its ligand LLT1, inhibits NK cell cytotoxicity by a mechanism involving the activation of acid sphingomyelinase (37). Our results show that the expression of CD161 on CD56^dim^ NK cells is decreased in CMV-seropositive donors compared with young CMV-seronegative donors. Little is known regarding the effect of CMV and aging on the expression of CD161 on NK cell subsets. The analysis of telomere length in NK cells has shown that the average telomere length in human NK cells decrease with age. In addition, the telomere length was significantly shorter in CD56^dim^CD16^+^ NK cells compared to CD56^bright^CD16^−^ NK cells from the same donor indicating that this subset represents more immature NK cells (67). In this study, it was shown that although CD161 can be expressed on CD56^bright^ and CD56^dim^ subsets, its expression is independent of the level of differentiation estimated by the telomere length (67).

The expression of CD161 in T lymphocytes and NK cells in PBLs from healthy children appears unrelated to CMV serostatus (68). On the contrary, it has been reported that NKG2C^+^CD56^dim^ NK cells, expanded in CMV-seropositive chronic hepatitis patients, have a significant decrease in the expression of CD161 and a significant increase in the expression of CD57 (69). In agreement with these data, our results show that the majority of NK cells do not coexpress CD57 and CD161, and that CMV seropositivity is associated not only with an increase in the expression of CD57 in CD56^dim^ NK cells but also with a decrease in the expression of CD161 in this subset. The decreased expression of CD161 in NK cells from CMV-seropositive NK cells parallels the finding that CD161 is decreased on CD56^+^ NKT-like cells in CMV^+^ subjects compared with CMV-seronegative donors (70). Interestingly, it has also been shown that the expression of CD161 in CMV-specific cytotoxic T lymphocytes is very low (71). Considering the inhibitory capacity of CD161 on NK and T lymphocytes cytokine production, these results support that the CMV-induced downregulation of CD161 receptor together with the expansion of polyfunctional response of CD57^+^ NK cells and T- and NKT-like lymphocytes (14, 30, 69, 72, 73), contribute to the proinflammatory environment observed in CMV-seropositive healthy individuals.

Recent reports have strengthened the significance of T-bet and Eomes in NK cell biology (47). In mice, T-bet and Eomes are necessary for maintenance of peripheral NK cells, their deletion in mature NK cells results in reversion to an immature phenotype (46). These transcription factors also modulate many NK cell effector functions, including cytotoxicity and cytokine production (74, 75). In human NK cells, T-bet and Eomes are differentially expressed on NK cell subsets (45), supporting that they can regulate different functions in different NK cell subpopulations. Our results confirm and extend these results showing that the levels of T-bet and Eomes are modulated in peripheral blood NK cell subsets representing different maturation stages, independently of aging and CMV serostatus. The expression of T-bet is lower in the CD56^bright^ NK cells than in the CD56^dim^ subset, whereas the expression of Eomes is higher in the CD56^bright^ NK cells. The CD56^dim^CD57^+^ subset shows higher levels of T-bet and lower levels of Eomes than the CD56^bright^ and CD56^dim^CD57^−^ NK cells. These results agree with the finding that not only the expression of T-bet and Eomes but also the expression of CD57 marker, modulate in parallel with the increase of KIRs on NK cells, but they do not differ in licensed or unlicensed NK cells (76), supporting that NK cell maturation but not NK cell licensing is dependent on T-bet and Eomes modulation. The significance of T-bet and Eomes in the maturation of other NK cell subpopulations is also supported by the demonstration that other subsets of NK cells display distinct patterns of expression of these transcription factors. Thus, a subpopulation of tissue-resident hepatic CD56^bright^ NK-cells, adapted to the tolerogenic liver microenvironment, with reduced proinflammatory potential, and characterized by the expression of CXCR6, express high levels of Eomes and low levels of T-bet, a phenotype virtually absent from peripheral blood (77, 78).

Very little is known on the expression patterns of T-bet and Eomes within human NK cell subpopulations in clinical situations. In this work, we have analyzed the expression of these transcription factors in two circumstances, such as CMV chronic infection and aging, which have a profound impact in NK cell differentiation.

Our results show that CMV seropositivity in young individuals associates with a significant increase in the percentage of CD56^dim^ NK cells expressing Eomes and a decreased percentage of T-bet^hi^ NK cells within the CD56^dim^CD57^+^ subset, suggesting that changes in the expression of these transcription factors are involved in CMV-induced remodeling of NK cells, characterized by the expansion of CD56^dim^CD57^+^ NK cells coexpressing NKG2C activating receptors (2, 14, 30, 60, 79). It has been reported that the level of expression of Eomes and T-bet is strongly reduced in NK cells from allogeneic hematopoietic stem cell transplantation recipients compared with healthy control subjects and that acute graft-versus-host disease and CMV reactivation are associated with further downregulation of T-bet (80) supporting the importance of these transcription factors in the generation and differentiation of NK cells and in the response against CMV after hematopoietic stem cell transplantation.

The analysis of the effect of age on Eomes expression showed a decreased expression in CD56^bright^ NK cells from CMV-seronegative middle age donors compared with CMV-seronegative young individuals, whereas in CMV-seropositive donors, its expression was significantly higher in the three NK cell subsets, in young donors compared with the middle age and old groups. The percentage of T-bet^hi^ NK cells was not significantly affected by age, independently of CMV serostatus. The effect of aging on the expression of transcription factors in NK cells is not known. It has been recently shown that impaired NK cell maturation in old mice is associated with a decreased expression of T-bet and Eomes in aged bone marrow NK cells. The use of bone marrow chimeras has revealed that the non-hematopoietic environment is responsible for the impaired maturation and function of NK cells, including the defective expression of T-bet and Eomes expression on NK cells (81). Considering that the defect in NK cell generation and in T-bet and Eomes expression in old mice is due to age alterations in non-hematopoietic environment, a better understanding of these non-hematopoietic factors involved in NK cell differentiation is required for the definition of new strategies aiming to improve NK cell function in the elderly.

It has been shown that the expression of T-bet and Eomes is increased in non-naïve CD8^+^ T cells from aged subjects and that this increase correlated closely with the levels of CD57 and KLRG1. In addition, it was shown that aging is associated with a decreased functionality of influenza virus-specific CD8^+^ T cells and increased expression of CD57, KLRG1, and T-bet (82), supporting that the increased expression of these transcription factors is related to the expansion of highly differentiated, senescent or exhausted, CD8^+^ T cells found in elderly individuals.

In conclusion, CMV latent infection has a profound impact on NK cells inducing significant changes in the expression of NK receptors, including the inhibitory receptors CD300a and CD161. T-bet and Eomes are differentially expressed on NK cell subsets defined by the expression of CD56 and CD57, and its expression is affected by CMV latent infection and aging, which can be involved in the age-associated changes observed in the differentiation and function of NK cells.

## Author Contributions

RS, CC, and AP designed the study. NL-S collected the data and performed the laboratory experiments. NL-S and FH performed the laboratory analysis. CC and NL-S performed the statistical analysis and wrote the draft. RT, BS-C, CC, and AP made significant conceptual contributions to the manuscript. RS, RT, and CC reviewed the final version of the paper. All the authors provided intellectual content and approved the final version of the paper.

## Conflict of Interest Statement

The authors declare that the research was conducted in the absence of any commercial or financial relationships that could be construed as a potential conflict of interest.
