# Supplementary material for: Effect of CMV and Aging on the Differential Expression of CD300a, CD161, T-bet, and Eomes on NK Cell Subsets
Source: Front Immunol. 2016 Nov 7;7:476. doi: 10.3389/fimmu.2016.00476 (PMC5097920; doi:10.3389/fimmu.2016.00476)
Supplement: Supplementary file 1 [file Image_1.PDF]

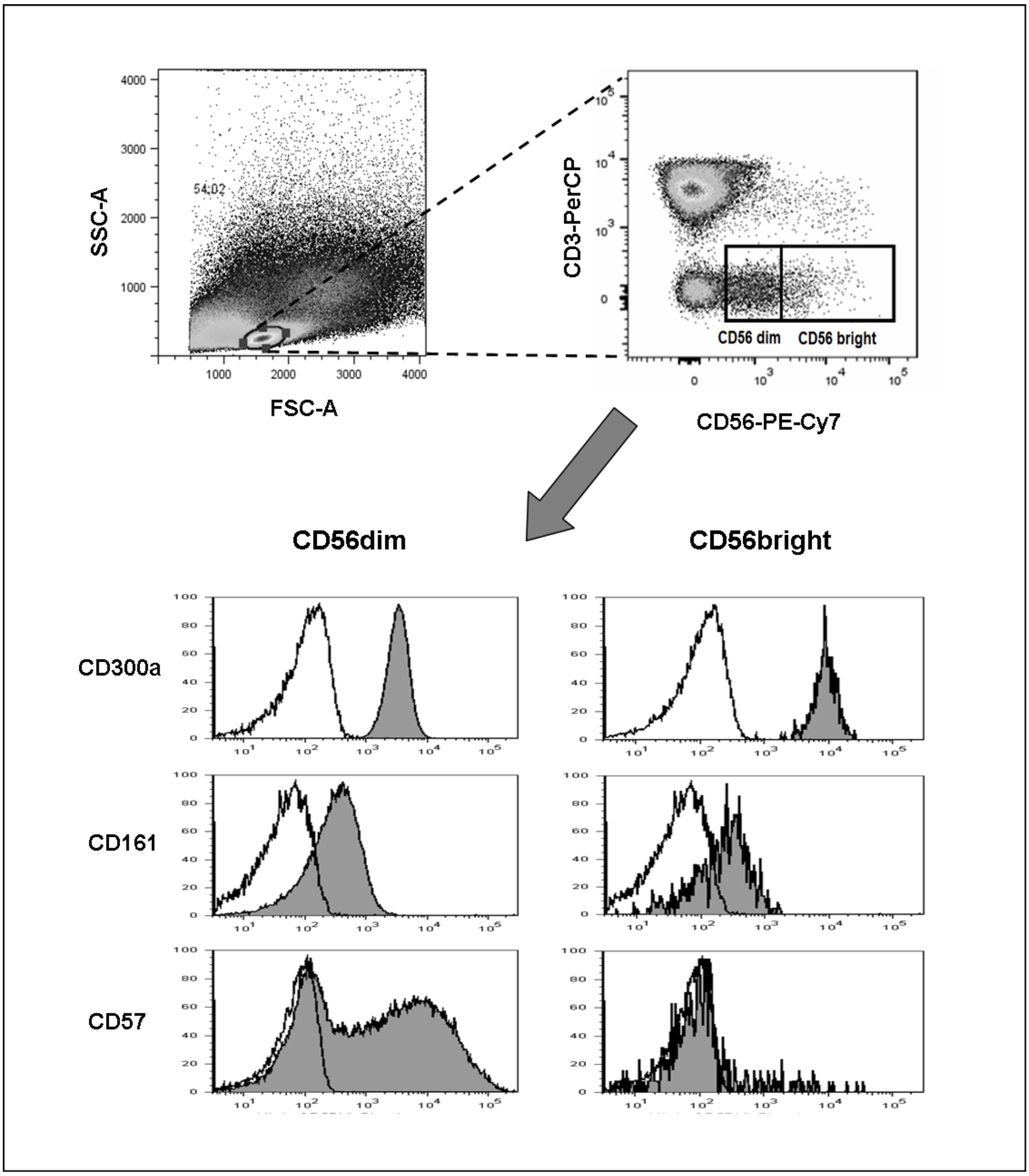

Figure S1. Gating strategy used for the analysis of CD57, CD300a and CD161 on NK cells by Flow Cytometry. Peripheral blood lymphocytes (PBLs) were selected using forward (FSC) and side scatter (SSC) detectors and $CD3^{-}CD56^{+}$ NK cells were gated from PBLs after single cells gating. CD57, CD300a and CD161 expression (measured as percentage) was determined on $CD56^{dim}$ and $CD56^{bright}$ NK cells.
