# Supplementary material for: Effect of CMV and Aging on the Differential Expression of CD300a, CD161, T-bet, and Eomes on NK Cell Subsets
Source: Front Immunol. 2016 Nov 7;7:476. doi: 10.3389/fimmu.2016.00476 (PMC5097920; doi:10.3389/fimmu.2016.00476)
Supplement: Supplementary file 2 [file Image_2.PDF]

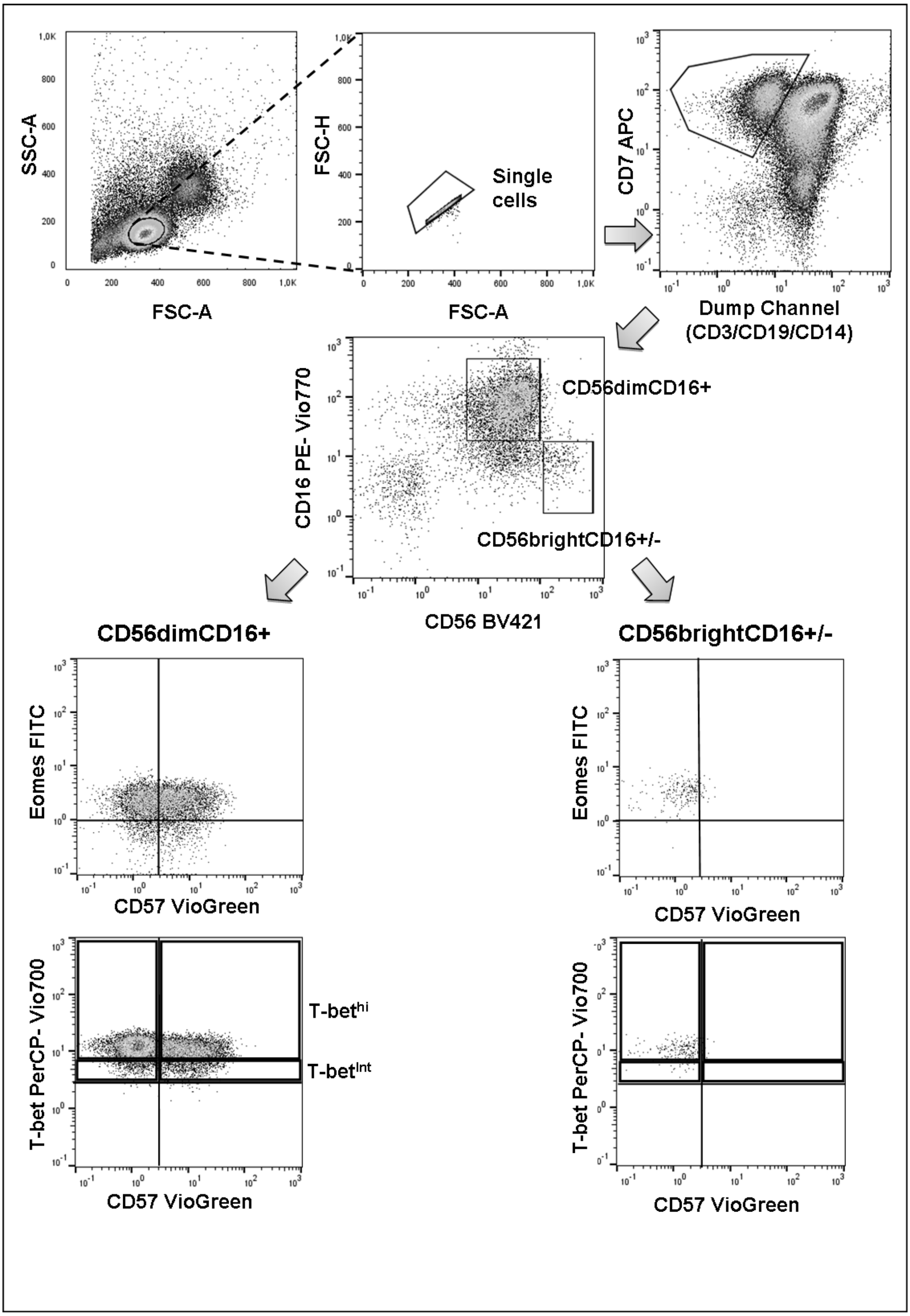

Figure S2. Gating strategy used for the analysis of Eomes and T-bet expression on NK cell subsets. $CD7^{+}CD3^{-}CD19^{-}CD14^{-}$ cells were gated of total PBLs, after isolating the single cells. NK cells subsets were selected according to expression density of CD56 and CD16 markers, followed by CD57 expression. Three subpopulations of NK cells were described: $CD56^{bright}CD57^{-}$, $CD56^{dim}CD57^{-}$ and $CD56^{dim}CD57^{+}$. The expression of T-bet and Eomes was measured in these NK subsets by Flow Cytometry. Two subsets of cells were defined according to the level of T-bet expression: T-bet^{hi} and T-bet^{int}.
